# Developing a Life Story Intervention for Older Adults With Dementia or at Risk of Delirium Who Were Hospitalized: Multistage, Stakeholder-Engaged Co-Design Study

**DOI:** 10.2196/59306

**Published:** 2024-09-27

**Authors:** Sarah J Flessa, James D Harrison, Roniela Turnigan, Megan Rathfon, Michael Chandler, Jay Newton-Small, Stephanie E Rogers

**Affiliations:** 1 MemoryWell Albuquerque, NM United States; 2 Division of Hospital Medicine Department of Medicine University of California, San Francisco San Francisco, CA United States; 3 Division of Geriatrics Department of Medicine University of California, San Francisco San Francisco, CA United States

**Keywords:** co-design, storytelling, dementia, delirium, older adults, person-centered care

## Abstract

**Background:**

Older adults with chronic or acute cognitive impairment, such as dementia or delirium, who are hospitalized face unique barriers to person-centered care and a higher risk for negative outcomes stemming from hospitalizations. There is a need for co-designed interventions adapted for these patients to the hospital setting to improve care and outcomes. Patient life storytelling interventions have demonstrated promise in enhancing person-centered care by improving patient–care team relationships and providing information to enable care tailored to individual needs and values.

**Objective:**

This study aims to engage patients, care partners, and clinical stakeholders in a co-design process to adapt an existing life storytelling model for use with older adults with dementia and at risk of delirium in the acute care hospital setting.

**Methods:**

We recruited patients with dementia or at risk of delirium who were hospitalized, their care partners, clinicians, and informaticists. A 3-stage co-design process that used a mixed methods data collection approach including in-depth interviews and surveys was completed. We used content analysis to analyze qualitative data and descriptive statistics to summarize quantitative data.

**Results:**

In total, 27 stakeholder informants (ie, patients, care partners, and interdisciplinary care team [IDT] members) participated. Stakeholders were unanimously interested in using patient life stories as a tool for hospital care through electronic health record (EHR) integration. Stakeholders shared potential topics for life stories to cover, including social support, information on patients’ key life events, and favorite activities. Participants provided insights into the logistics of integrating life stories into acute care, including interview arrangement, story-sharing methods, and barriers and facilitators. IDT members shared preferences on EHR integration, resulting in 3 co-designed mock-ups of EHR integration options. Stakeholders shared ways to optimize future acceptability and uptake, including engaging with the care team and promoting awareness of life stories, ensuring suitability to the acute environment (eg, distilling information in an easily digestible way), and addressing concerns for patient capacity and privacy (eg, engaging care partners when appropriate). Thoughts on potential impacts of life stories were also elicited, including improving patient- and care partner–IDT member relationships; humanizing patients; increasing clinical team, patient, and caregiver satisfaction; and enabling more specific, tailored care for patients with dementia and at risk of delirium.

**Conclusions:**

This study resulted in a co-designed life storytelling intervention for patients with dementia and at risk for delirium in an acute care hospital setting. Stakeholders provided valuable information to ensure future intervention acceptability and uptake, including potential benefits, facilitators, and challenges in the acute care setting.

## Introduction

### Background

Between 25% and 40% of patients aged ≥65 years have acute or chronic cognitive impairment during a hospitalization, which can greatly affect their experience, care, and ability to communicate [1]. This impairment can be a chronic deteriorating impairment as is seen in Alzheimer or other dementias, or it can be an acute and likely reversible impairment which happens in hospital-induced delirium. For older adults with dementia or at risk of delirium, hospitalizations are often dehumanizing and disorienting [2,3]. No matter if it is a chronic cognitive impairment or an acute delirium episode, it is often difficult for these patients to communicate their needs and preferences in a new environment such as the hospital.

These challenges, coupled with a hospital’s limited set of resources, tools, and time to provide person-centered care can contribute to loss of patient dignity and autonomy [2,4] and result in negative quality and clinical outcomes, including increased restraint use, longer hospital stays, and higher hospital mortality compared to patients without cognitive impairment [1,5-7]. In addition, hospital interdisciplinary care team (IDT) members report feeling moral distress and low satisfaction when they are unable to provide high-quality, person-centered care for patients with dementia and delirium [8]. Care partners for older adults with dementia and delirium experience increased caregiver strain and negative health outcomes from the stress of caregiving [9-13].

Addressing the complexities associated with high-quality, person-centered care for patients with dementia or at risk of delirium requires co-designed solutions that accommodate the workplace demands of the hospital IDTs and prioritize the well-being and individual needs of patients and care partners. Co-design is a dynamic process, endorsed by leading health agencies [14], that involves active engagement of stakeholders in intervention development to produce sustainable, acceptable interventions [15,16].

By acknowledging the unique demands and requirements of multiple clinical roles within the IDT, we can develop interventions that fit into the existing resource-limited care context, resulting in more effective and efficient health care delivery. Engaging IDT members in intervention development can improve their satisfaction and ultimate uptake of the intervention [16]. Co-design has been used to improve the use of electronic information exchange tools (eg, patient portals) by eliciting patient, care partner, care team, and other stakeholders’ perspectives on barriers and facilitators to engagement with these tools [17].

In addition, co-designing with older adults with dementia or at risk of delirium presents an opportunity to understand their needs and wishes [18] but comes with logistical and ethical challenges regarding capacity to consent and participate in research. There is a paucity of research including patients with dementia or at risk of delirium who were hospitalized, in co-design. A recent review identified only 8 published co-design studies in the acute care setting, and none focused specifically on patients with dementia or at risk of delirium [19]. Interviewing the older adults in the presence of their care partner can help with communication [18].

Given the unique barriers to providing person-centered care for older adults facing cognitive issues who were hospitalized, co-designed interventions adapted to this setting and population are sorely needed. Life stories and similar narratives have emerged as a powerful strategy to enhance person-centered care by improving patient–care team relationships and communication. Life stories include information about patients’ values, preferences, and personal experiences that enable the delivery of personalized care aligned with patients’ unique needs. Research on life stories and similar narratives has shown improvements in patient–care team relationships and communication, as well as patient well-being [20-24]. However, life stories have yet to be tailored to the acute care setting for older adult patients with dementia and at risk of delirium.

### This Study

Because of negative hospital experiences (eg, experiencing confusion and disorientation), communication challenges, and barriers to person-centered care in the hospital, these populations may similarly stand to benefit from patient life storytelling. The aim of this study was to describe a stakeholder-engaged co-design process to adapt an existing life storytelling model for older adults with dementia or at risk of delirium in the acute care hospital setting.

## Methods

### Study Design

The co-design process involved using a mixed methods data collection approach including in-depth individual interviews and survey.

### Setting

This study took place in the Acute Care for Elders (ACE) Unit at the University of California San Francisco (UCSF) Medical Center between May and December 2022. The UCSF ACE Unit provides a specialized acute care environment for older adult patients to promote independence and function and prevent complications such as delirium that are common for older adults who are hospitalized [25].

### Ethical Considerations

This study was reviewed by the Advarra Institutional Review Board (Pro00056445) and deemed exempt in accordance with the US Department of Health and Human Services regulations at Title 45 Code of Federal Regulations Part 46. Participants provided written informed consent. Patients were able to participate if their legally authorized representative or surrogate provided written informed consent. Participants were remunerated with Visa gift cards for participating; US $20 for surveys and US $50 for qualitative interviews. All data were deidentified before analysis.

### Intervention Development and Description

MemoryWell life stories, which have been used primarily in long-term care settings since 2017, were developed by expert interviewers (JN-S and MC) with backgrounds in journalism. They developed the life story questionnaire drawing from evidence-based, dementia-friendly interview strategies [26] as well as user experience at long-term care and palliative care settings at 45 sites in more than 20 states. MemoryWell’s life stories have demonstrated improved experiences for patients, care partners, and care staff; in one 30-story pilot at a skilled nursing facility (SNF), patients and family members reported feeling better understood because of the life story, and family members reported improved relationships with care staff [27]. Staff reported increased satisfaction and ability to care for patients considering their needs and wishes after life story intervention [27].

Life stories include information about patients’ life stages (eg, childhood and early adulthood), achievements, challenges, religion and spirituality, favorite activities, and social support. MemoryWell’s life storytelling model consists of a 30- to 45-minute interview conducted by trained interviewers with a patient or their care partner, or both together. The life story interview format is dependent on the patient’s cognitive capacity, care needs, and family preference and availability. When possible, patients participate in all or part of the life story interview independently, and the interviewer can contact their care partner to provide additional information if needed. Otherwise, the interview is conducted with the patient and a care partner present. Questions are addressed to the patient, and the care partner fills in and supports the interview as needed. For patients who are unable to recall or communicate their life stories or whose care needs interfere with the ability to give an interview, a care partner can provide the interview on their behalf. This interview is audio recorded and automatically transcribed. On the basis of this interview, a professional writer writes an approximately 500- to 700-word life story narrative, which takes approximately 1 to 2 hours. The story is then reviewed by an editor for clarity, which takes about half an hour, before being shared with the family and care team. The story is intended to inform care for this particular patient. This study’s goal was to adapt MemoryWell’s SNF life story intervention to the acute care setting for older adults with dementia or at risk of delirium, reflecting setting differences in patient continuity, workflow, and time constraints.

### Participants and Recruitment

#### Patient and Care Partner Recruitment

We used purposeful sampling [28] to identify eligible patients and care partners to participate in in-depth interviews for the co-design process. Eligible patients were English-speaking adults aged ≥65 years, admitted to the ACE Unit, with a diagnosis of dementia documented in their medical chart or a positive AWOL score [29], a validated hospital admission-screening tool that determines the risk of acquiring hospital delirium. The AWOL score uses a person’s age (A), ability to spell “world” backward (W), orientation to environment (O), and a nurse assessment of illness severity (L) to determine not only an increased risk of delirium during their hospitalization but also it gives the medical team general information about a patient’s current cognitive status (whether it is acute delirium or a more chronic cognitive issue, although it does not distinguish the difference). A positive score indicates an increased risk for hospital delirium. English-speaking care partners aged ≥18 years of eligible patients were also invited to participate. If a care partner spoke English but the patient did not, care partners were invited to participate. Non-English speakers were not enrolled due to constraints in translation resources for consent and interviews.

Eligible patients and care partners were identified daily through the electronic health record (EHR). Eligible patients and care partners were then invited to participate in the study in person during their hospitalization or via telephone shortly after their hospital discharge.

#### IDT Members and EHR Informaticist Recruitment

Inpatient IDT members were eligible if they delivered care to an eligible patient in the ACE Unit. Using purposive sampling [28], inpatient IDT members were invited to participate via email. To address different clinical perspectives, IDT members from a variety of roles on the ACE Unit were invited, including geriatricians, hospitalists, physical therapists, chaplains, social workers, case managers, nurse practitioners, registered nurses, and pharmacists. To explore the potential utility of life stories in the patients’ care transitions after hospitalization, eligible outpatient geriatric primary care and postacute care providers were invited by email. These participants included physicians, geriatricians, nurse practitioners, and a SNF medical director.

Eligible EHR informaticists and programmers from the UCSF APeX Enabled Research (AER) program were invited to participate. APeX is the name of the Epic EHR used at UCSF. The AER program supports the use of APeX for faculty-initiated research projects and provided consultation and technical services for this project [30].

### Data Collection and Co-Design Process

**Figure 1:**
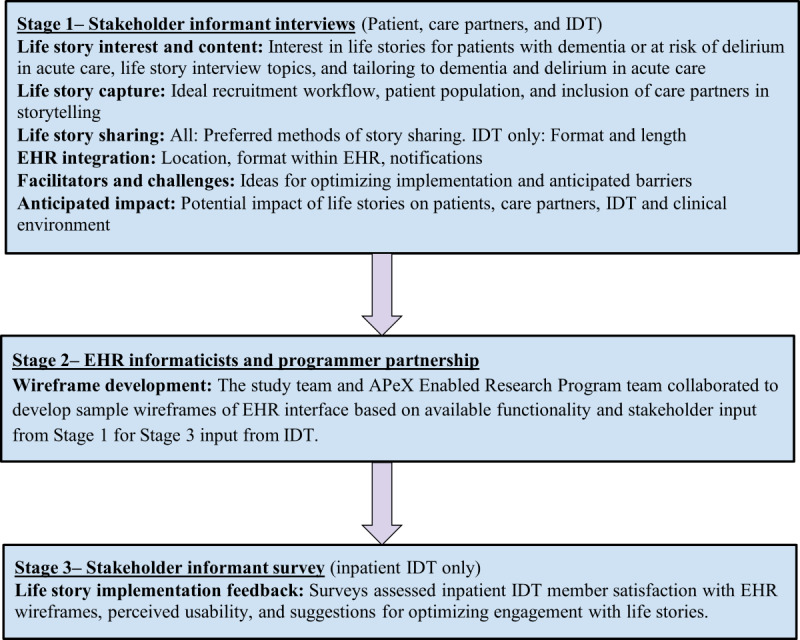
Co-design of life storytelling intervention for patients with dementia or at risk of delirium who were hospitalized, in acute care. EHR: electronic health record; IDT: interdisciplinary care team.

Figure 1 demonstrates our 3-stage co-design process with stakeholders and methods used.

#### Stage 1: Stakeholder Informant Interviews

Stage 1 involved in-depth qualitative interviews with stakeholder informants. We developed a study-specific interview guide, based on our previous experience interviewing patients with dementia in long-term care, to assess the feasibility and acceptability of integrating life stories into acute care for patients with dementia or at risk of delirium (Multimedia Appendix 1). At the beginning of each interview, stakeholders were given an overview of the purpose of the study which included the overarching goal of developing a life story intervention for implementation in the acute care setting. The questions explored the stakeholder’s level of interest in life stories and anticipated impacts on care. Interviews elicited preferred topics for life stories as well as content that stakeholders did not want to be included in life story interviews. We also explored stakeholders’ views on how life stories could be implemented into hospital clinical practice such as preferences on the life story interview process, including timing (eg, in relation to hospital stay and time of day) and method (eg, in-person vs remote and who participates in the interview). Barriers and facilitators to life story implementation and workflows were also assessed. Inpatient and outpatient IDT members were also asked about their preferences for integrating life stories into the EHR. Questions included life story format, preferred location within EHR, and strategies for integrating life stories into clinical workflows. All interviews were digitally recorded.

#### Stage 2: EHR Informaticists and Programmer Partnership

Data from stage 1 interviews were then used to inform the design of the life story intervention and implementation workflow in partnership with informaticists and EHR programmers. We identified opportunities for feasible and functional EHR integration. The AER team created sample wireframes (2D illustrations of the EHR interface) to demonstrate EHR integration options to stakeholders.

#### Stage 3: Stakeholder Informant Survey

Stage 3 involved asking stakeholder informants to provide additional feedback and insights on the intervention and implementation workflow using the wireframes as potential examples. We developed a survey for inpatient IDT members using study-created measures and measures adapted from the American Customer Satisfaction Index to assess their satisfaction with EHR wireframes, perceived usability, and suggestions for optimizing engagement. Measures were rated on a scale of 1 to 10, with 1 being the least desirable and 10 being the most desirable (eg, convenience of accessing: 1=very difficult and 10=very easy; satisfaction: 1=very dissatisfied and 10=very satisfied). Surveys were administered via email using SurveyMonkey (SurveyMonkey Inc) [31].

### Analysis

Qualitative interviews (stage 1) were deidentified and professionally transcribed verbatim. We used a combination of qualitative and quantitative content analysis for interview data [32,33]. Transcripts were reviewed independently by 2 members of the research team (SJF and JDH). We first used qualitative content analysis to systematically examine the transcripts to obtain a condensed understanding and description of the content [33]. We used a data-driven (inductive) approach to analysis whereby open coding was performed to identify salient and elevated topics of importance within the data set. Reviewers (SJF and JDH) met to define and refine coding categories to ensure trustworthiness throughout the analysis. Negotiated consensus was used to resolve disparities and coding categories [34]. We then organized codes around key study questions: interest and content, story capture, story sharing and EHR integration, anticipated impact, and facilitators and challenges to implementation. Quantitative content analysis was then performed to count coding categories. This was conducted for the purpose of providing a more detailed assessment of how frequently certain codes or themes were mentioned. Data analysis was managed in Dedoose (SocioCultural Research Consultants, LLC) [35]. Stage 3 surveys were analyzed using descriptive statistics.

## Results

### Stage 1: Stakeholder Informant Interviews

Of the 27 individuals interviewed, 3 (11%) were patients, 6 (22%) were care partners, and 18 (67%) were IDT members. Table 1 shows stakeholder *interest* in life stories and potential *topics to include* in life stories for patients, care partners, and IDT members. All stakeholders were interested in implementing patient life stories in the acute care setting. Care partners and patients focused on wanting to share who the patient is as a person outside of their current state, including reviewing the patient’s life stages (eg, education, former occupations, and places they have lived) and favorite activities. Several IDT members indicated that while they suspected certain preselected topics could be useful, they would want to know what patients and care partners prefer to share in each story. All IDT members wanted to know about the patient’s social support, as this was deemed to be critical knowledge for tailoring care plans.

**Table 1:** Life story level of interest and potential topics to include^a^.

			Total (N=27), n (%)		Patients (n=3), n (%)		Caregivers (n=6), n (%)		IDT^b^ (n=18), n (%)
Interested in life stories for acute care			27 (100)		3 (100)		6 (100)		18 (100)
**Topics^c^**									
	Life stages	23 (92)		1 (100)		6 (100)		16 (89)	
	Social support	23 (92)		1 (100)		4 (67)		18 (100)	
	Spirituality	6 (24)		0 (0)		2 (33)		4 (22)	
	Hobbies and activities	20 (80)		1 (100)		5 (83)		14 (78)	
	Preferred foods	5 (20)		0 (0)		3 (50)		2 (11)	
	Legacy and achievements	11 (44)		0 (0)		3 (50)		8 (44)	
	SDOH^d^, mental health, and trauma	14 (56)		0 (0)		2 (33)		12 (67)	
	Care preferences	7 (28)		0 (0)		1 (17)		6 (33)	
	What brings joy	11 (44)		1 (100)		2 (33)		8 (44)	

^a^Where data do not equal 100%, data are missing due to nonresponse.

^b^IDT: interdisciplinary care team.

^c^Only one patient answered about topics to include, so N=25 for these rows and n=1 for the patient.

^d^SDOH: social determinants of health.

### Life Story Interview Process

#### Patient and Care Partner Perspectives on Life Story Interview Timing and Format

##### Timing of Life Story Interview

Most patients and care partners indicated they would be willing to complete a life story interview during hospitalization if the interview would not interrupt daily activities, including medical procedures or favorite television programs. In addition, 1 care partner said that there was too much going on while the patient was hospitalized, so it would be preferable to capture stories before hospitalization.

##### Life Story Interview Format

Patients and care partners responded that in-person or virtual interviews were preferred; 1 care partner noted that because internet connectivity was poor in some patient rooms, Wi-Fi hot spots would need to be provided to conduct Zoom (Zoom Video Communications, Inc) interviews.

#### IDT Member Perspectives on Life Story Interview Timing and Format

##### Timing of Life Story Interview

Themes from IDT members regarding life story interview scheduling include challenges navigating short lengths of stay, ensuring patients are medically stable, and finding a time of day that fits the hospital workflow and patient needs. To ensure the story is used within short lengths of stay, IDT members suggested collecting stories as soon as possible once the patient is out of the emergency department on the hospital floor. Life story interviews must fit the clinical workflow, in between medication administration, testing, specialist visits, procedures, and mealtimes. The optimal time between these tasks, when patients are most alert, was noted to be between 10 AM and 5 PM.

##### Life Story Interview Format

IDT members highlighted that life story interviews must be responsive to COVID-safety considerations as well as technology and visual and hearing needs of patients and families. They cautioned that Zoom interviews may be difficult for patients if they have issues with hearing or vision. For patients with cognitive impairment, they recommended care partners be involved in life story interviews. Care partners supported having an interviewer from outside of the care team conduct the life story interview and write the life story if they were made aware that the interviewer was from an outside organization, noting that professional writers would be “the right people for the job.” They felt MemoryWell conducting interviews would save time and resources for the care team while helping fill in information gaps; MemoryWell was described as a “bridge to patients for the care team.”

### Life Story Sharing

#### Life Story Sharing With Patients and Care Partners

Patients and care partners suggested a variety of methods to personally receive their stories after they had been completed, including via email, paper copies, and UCSF’s patient portal, MyChart.

#### Life Story Sharing With IDT Members

##### Life Story Format

Most IDT members indicated that they would prefer a story format that includes both paragraphs and bullet points. Bullet points were perceived to skim information quickly when IDT members have limited time while paragraphs can offer more detailed context. IDT members prioritized ease of reading, and several suggested that communicating valuable information in a concise manner was more vital to ensuring use than the exact length of the story. IDT members emphasized the importance of keeping the stories brief to ensure that they would not get overwhelmed and avoid reading stories. Most suggested 1 to 2 pages and others preferred even briefer stories at just 2 to 3 paragraphs or 350 words.

##### EHR Integration

IDT members emphasized the importance of ease of access to life stories in the EHR to ensure they could incorporate life stories into their workflow. Most IDT members suggested integrating life stories into the social history section because the life story content intuitively aligns with this section and most clinicians would be able to find it. Further, the social history EHR section automatically pulls into notes and is a static location that can be viewed as an inpatient or outpatient IDT member. Others suggested locations that would be visible immediately upon opening the patient’s chart or integrating into the EHR’s Daily Rounds tab. IDT members stressed avoiding information overload in clinical notes or other important EHR pages and suggested potentially storing a summary in some sections (ie, social history) with an option to click to another location for the full life story.

IDT members said that making the story easy to find is the most significant priority, preferable to creating alerts or notifications that a story is available; several suggested an icon next to the patient’s name that would change colors to indicate that the story was available. Other suggestions included only flagging for especially critical information, such as trauma history, through a pop-up notification.

### Anticipated Impact of Life Story Integration Into EHR and Patient Care

#### Overview

Themes and exemplary quotes about the anticipated impact of life stories can be found in Table 2.

**Table 2:** Anticipated impact of life story integration into EHR^a^ and patient care.

Stakeholder, theme, and theme description		Quotes
**Patients and care partners**		
	Theme 1: Life stories can help the care team see patient as a person beyond their medical issues.	“One doctor came in and he was very kind of brusque and fast talking, didn’t even really look that much at us at the beginning...I felt like saying, hey...my mom is a person, not just a patient, she’s a person.”
	Theme 2: Life stories may support patient and IDT^b^ relationship building.	“I think the best would be if the doctor can have the capacity to take on this patient,...and have that kind of personal time to get to know the patient. Because caring, the feeling is so complicated. There’s magic to it, and it can only come from like a true...kind of collaboration...when a doctor makes a connection with a patient.”
	Theme 3: Life stories can provide context that could help prevent discrimination based on age and race.	“Not enough credit is given to the elderly to be able to think and express themselves. That throws them into a depressed state of mind, thinking that it’s over for me. But if they are involved and respected, it’s a better transition.” “I think they would treat her differently. It’s unfortunate that when people perceive you, the outside of you, they have a limited view of what you’re really like as a person...For example, people of other cultures tend to look at African Americans as not being knowledgeable, not being experienced with the other parts of the world.” “I just think that... people have a really limited view of older people. I think especially with older people that if they know... she’s really sharp and she’s usually really active, I think that... it would make a difference—if they actually wanted to know. I just have a lot of doubt about them really actually wanting to know.”
**IDT members**		
	Theme 1: Life stories could help improve person-centered care by (1) humanizing patients, (2) fostering patient- and care partner–IDT relationships and communication, and (3) enabling care tailored to what matters the most to patients.	“I think that the humanizing element is really important. In the hospital specifically... there are many dangers of having a narrow track mind, and we want people to fit in the narrow track... and so the more ways that we can bring out the individuality, the intriguing parts of a person’s story, I think that can soften our approach and actually make it easier to do our jobs.” “You have all the tests and you have all the medications available, but really don’t have the time to sit down and know them. So maybe working as a team would be helpful, where maybe the care team there could be a champion saying okay, this is a person who is going to make sure this person’s wishes are done, and inform everybody in the team. It could be an email, as you said, like where the case manager sends an email. Oh, just wanted to let you know this other thing is on the person’s life story, just for the team to know.” “Our providers are working in an increasingly busy care environment. They want more support, because we’re being asked...to see more patients with less support...it is a moral distress as an inpatient provider. You want to spend time with patients, you want to get to know them. So the value proposition for this is like, this is a tool that allows your providers to get to know your patients, starting off instead of at first base on third base, with all this information about them in a very easy way that’s user friendly, that’s right in front of them, that’s readable, where you don’t have to spend time as a provider sifting through the chart.”
	Theme 2: Life stories could be especially helpful for caring for patients with CI^c^ or delirium.	“It gives us things to talk about with the patients, and it’s helpful in cases of delirium or dementia to know what was important to that person so that we can kind of help ground them and give them more context, just make them feel less lost in the alien atmosphere of being in the hospital.” “When you do medical interventions to this type of patient it can be very challenging sometimes because of their behavior. So I think it gives an in to the clinicians of how to soothe the patient and making sure oh, this patient can still make a little bit of decision with guidance. If we have that in the life story of the medical records, then we know already...the patient is not a stranger to us...we already know the patient’s background.” “I think it’s extremely important to connect with your patient, especially because people with dementia are usually older and frail and scared in the hospital. And they do best, a lot of times they do best when they’re around familiar surroundings, which they can’t be around, so familiar conversation always helps provide some comfort.”
	Theme 3: Life stories help improve stakeholder satisfaction.	“And knowing more about who our patients are and what they’ve done with their lives and what was important to them really humanizes them and I think facilitates staff to have greater empathy for their situation. Frankly, I think it improves care for everybody. I think the staff feels more satisfied, feeling like they know who they’re taking care of.” “I imagine that in general people like to feel known and seen for who they are.” “I feel like families often feel more comfortable with their loved one being in the hospital if they feel like we know their loved one well, we care about trying to make them comfortable.”

^a^EHR: electronic health record.

^b^IDT: interdisciplinary care team.

^c^CI: cognitive impairment.

#### Patient and Care Partner Themes About the Anticipated Impact of Life Stories

##### Theme 1: Life Stories Can Help Care Team See Patient as a Person Beyond Their Medical Issues

Patients and care partners said that life stories could be helpful in encouraging the IDT to see the patient as a full person.

##### Theme 2: Life Stories May Support Patient and IDT Relationship Building

Care partners expressed the importance of care teams taking the time to build genuine relationships with patients and that life stories could help foster these relationships by helping IDT members get to know their patients.

##### Theme 3: Life Stories Can Provide Context That Could Help Prevent Discrimination Based on Age and Race

Care partners also shared that life stories could help prevent ageism and discrimination, arguing that if the IDT understood patients’ life experiences and social context, they could be less quick to judge them based on their age or race. They also expressed that life stories could help with suboptimal care team communication and rapport.

#### IDT Member Themes About the Anticipated Impact of Life Stories

##### Theme 1: Life Stories Could Help Improve Person-Centered Care by (1) Humanizing Patients, (2) Fostering Patient- and Care Partner–IDT Member Relationships, and (3) Enabling Care Tailored to What Matters Most

IDT members said that knowing more about their patients as people could help humanize the patients. They expressed that in a setting with limited time and background information on patients, life stories could be a valuable *time-saver* in jump-starting the patient- and care partner–IDT relationship by providing context for building rapport and communication. Several IDT members also said that giving social and cultural context and knowing about trauma history and root causes of social needs can help “decrease bias and promote respect and cultural competency.”

IDT members expressed that life stories could be beneficial for tailoring their care around “*what matters most* to the patient.” Further, knowing about family dynamics and if there is a designated health care proxy can help the care team know whom to involve in decision-making. Knowing about previous functional status and current social support can help optimize care and discharge planning. IDT members expressed that providing better person-centered care could lead to better patient outcomes, such as reduced delirium and readmissions.

##### Theme 2: Life Stories Could Be Especially Helpful for Caring for Patients With Dementia and Delirium

IDT members said that information from life stories could be especially helpful for patients with dementia or at risk of delirium by helping orient patients in an unfamiliar, often scary environment. This could help prevent the use of physical and chemical restraints in response to challenging behaviors. Furthermore, when these patients are unable to speak for themselves, a life story could help document important information and give them a voice in the medical record.

##### Theme 3: Improved Satisfaction for Each Stakeholder

IDT members shared that building better relationships and providing optimized person-centered care could lead to satisfaction for patients, care partners, and IDT. The story can be given as a gift for the family, which offers a chance to reflect on the patient’s life during a difficult time. IDT members anticipated that patients and care partners could feel seen and heard by someone in the medical setting taking the time to listen to and document their stories. IDT members explained that they often feel moral distress in not being able to provide the care that they want to and that life stories could help them take a step back and remind them of the meaning of their work.

### Facilitators and Challenges to Implementation

Table 3 shows IDT member–reported potential barriers and facilitators to life story implementation. IDT members emphasized the importance of training the full IDT around life stories to ensure life story use by everyone who cares for the patient. They also raised concerns about the time constraints within acute care and the necessity for life stories to be easily accessible. IDT members said that while patients with dementia and delirium could be uniquely well-suited for a life story intervention, care must be taken to adapt to patient capacity, involve care partners when appropriate, and ensure understanding of how information is being shared and with whom.

**Table 3:** IDT^a^-reported potential implementation barriers and facilitators.

Barriers and facilitators		Description of barriers and facilitators
**Engaging care team and awareness of life stories**		
	Potential barriers	Need cultural buy-in to treat the whole person Life stories must be available for enough patients to become a part of the regular workflow Some specialties may be less likely to want to engage with life stories (eg, surgery and pharmacy) or see components as outside of the scope of their role (eg, social determinants of health)
	Potential facilitators	Providing training regarding (1) the existence of life stories, (2) how to access stories, and (3) the potential benefits of reading life stories Some specialties may be more apt to see the benefit of life stories (eg, geriatricians well-versed in dementia and delirium care)
**Suitability to acute environment**		
	Potential barriers	Short length of stay Story may not be a priority in comparison to acute needs Resource intensive
	Potential facilitators	Distill information into an easily digestible format Must be easily accessible in EHR^b^
**Concerns for patient capacity and privacy**		
	Potential barriers	Privacy concerns: some patients may not want to share personal information, especially for populations at risk of potential legal system involvement (eg, undocumented patients) Ensuring patients are aware all IDT members can access life story in EHR Patient capacity to consent and communicate is dependent on cognitive impairment and illness severity
	Potential facilitators	Engaging with care partners as appropriate Working with patients to find an appropriate time to conduct life story interviews within day and stay Patients with dementia and at risk of delirium stand to benefit the most

^a^IDT: interdisciplinary care team.

^b^EHR: electronic health record.

### Stage 2: EHR Informaticists and Programmer Partnership

**Figure 2:**
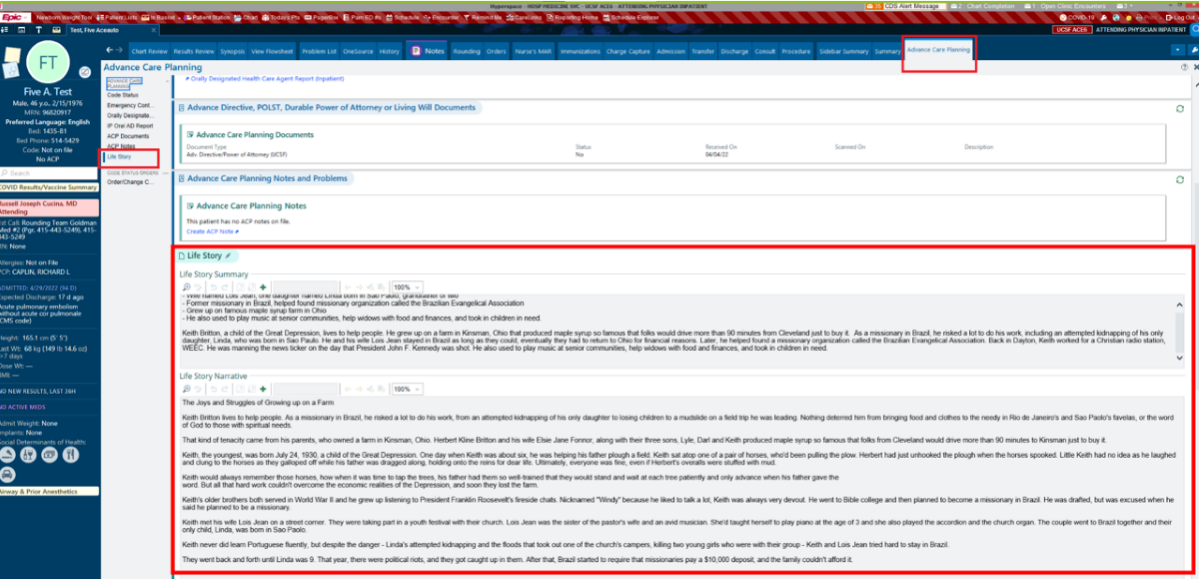
Advance Care Planning (ACP) tab. Life story summary and narrative on ACP tab. This page summarizes all ACP discussions and documents.

**Figure 3:**
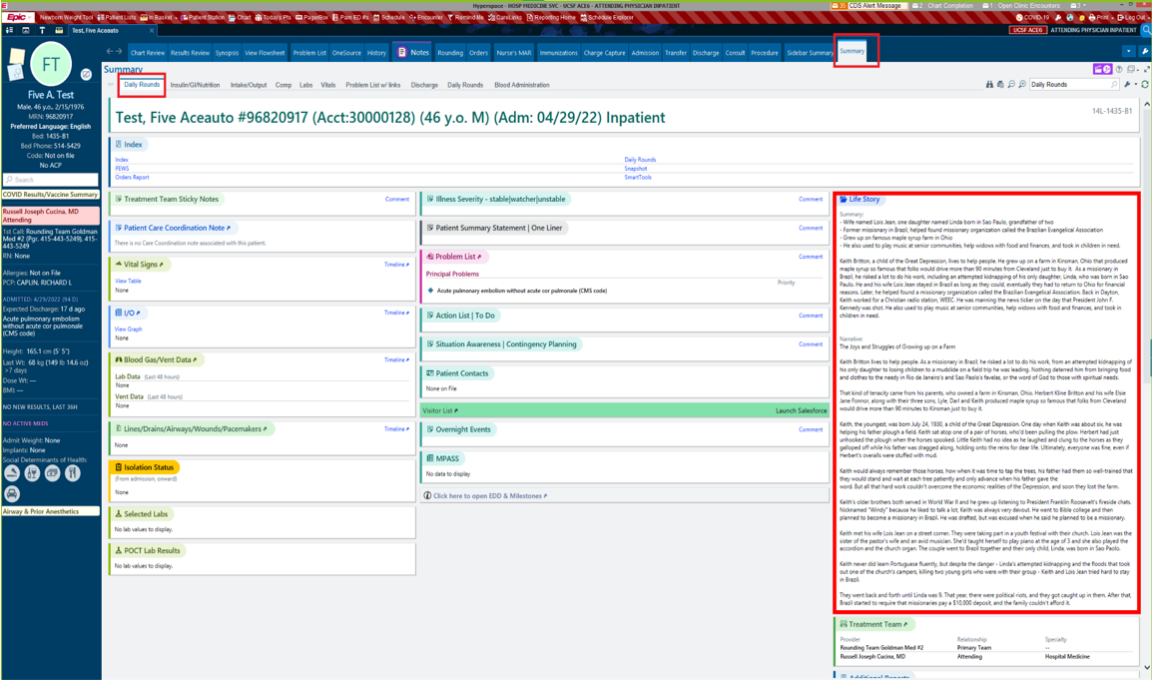
Daily Rounds tab. Life story summary and narrative on the Daily Rounds tab. This page is used to quickly collect basic clinical information before rounds.

**Figure 4:**
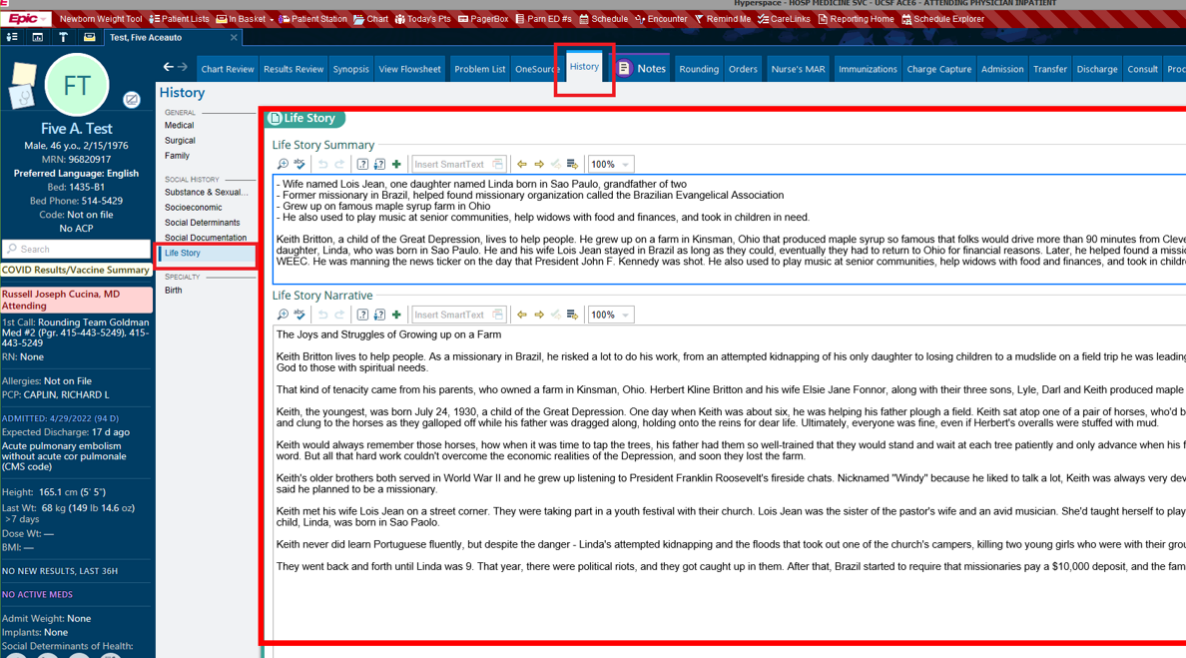
Social History section. Life story summary and narrative in the Social History section of overall History tab. This section is used to input information regarding the patient's social history.

We partnered with 3 members of the UCSF AER team who provided insights into feasible EHR integration. Moreover, 3 sample wireframes focusing on available functionalities, intended behaviors, and space allocation with prioritization of content were created incorporating options of how the life story would look in the EHR. Figures 2-4 present these wireframes in 3 potential EHR locations, including the Daily Rounds tab (used to quickly collect basic clinical information before rounds), Advance Care Planning (ACP) tab (which summarizes all ACP discussions and documents), and Social History section (the section to input a patient’s social history).

### Stage 3: Stakeholder Informant Survey

Stage 3 involved asking inpatient IDT stakeholder informants to provide feedback on EHR wireframes and life story implementation, including perceived usability and suggestions for optimizing engagement. Table 4 presents findings from the stakeholder informant survey, where 10 inpatient IDT members were surveyed with a 100% response rate. The Daily Rounds tab was rated highest on convenience of access and satisfaction (out of 10), followed by the Social History and ACP sections, respectively. The Daily Rounds tab was the most common top choice, followed by Social History. The majority (7/10, 70%) of IDT members responded they would review the story for all patients.

**Table 4:** Stakeholder informant survey of 3 sample EHR^a^ wireframe examples (N=10).

Location	Daily Rounds tab	Advance Care Planning tab	Social History section
Convenience, mean (SD)	8.3 (1.6)	5.5 (1.7)	5.5 (2.1)
Satisfaction, mean (SD)	7.7 (1.8)	5.7 (1.8)	6 (1.9)
Respondents who ranked top choice, n (%)	7 (70)	1 (10)	2 (20)
Respondents who ranked second choice, n (%)	1 (10)	2 (20)	7 (70)

^a^EHR: electronic health record.

### Outputs of Co-Design Process: Co-Designed Life Story and EHR Wireframe Selection

#### Co-Designed Life Story for Acute Care

On the basis of the feedback from stages 1 to 3, the resulting Life Story section of the wireframe was divided into 2 sections: Life Story Short-Form and Life Story Narrative. The Life Story Short Form is intended to provide concise information about the patient’s life story if a clinical team member is time-constrained. The The Life Story Short Form is formatted in bullet points and short-form questions were selected based on stakeholder input to include information deemed most readily applicable to acute care, including evidence-based commonly unmet needs for patients with dementia or at risk of delirium (eg, boredom, discomfort, and loneliness) [36,37], as well as what matters to align with the Age-Friendly Health Systems framework [38]. These short-form unmet needs questions are at the beginning of the co-designed life story interview guide and the life story interviewer outlines the responses in bullet points to allow for immediate sharing. Topics included social support, previous occupation, favorite entertainment (eg, television, reading, and music) and foods, religion or spirituality, what a good day at home might entail, and what matters most to the patient.

The Life Story Narrative is approximately 400 to 500 words and in paragraph format. This is written by a professional writer based on the life story interview recording and transcript and takes approximately 1 to 2 hours to write. It builds upon the summary to provide a rich description of the patient’s life experiences and covers life stages, achievements, challenges, and other information the patient or care partner wanted to share that is not covered in the summary. This narrative form is intended to provide a longer summary in case clinical team members want to spend more time getting to know a patient on a less busy clinical day.

#### EHR Wireframe Selection

After stages 1 to 3, both the Daily Rounds (Figure 3) and the Social History (Figure 4) locations were chosen for future piloting and EHR integration. These 2 options provided a static location that can be viewed in both the inpatient and outpatient context (Social History) and for ease of daily inpatient access (Daily Rounds and Social History pulling into notes), thus giving more opportunities for clinical care team members to read the stories in their usual daily clinical workflow. The Daily Rounds tab would include the Life Story Short-Form section for quick access, while the Social History would include the full life story (both the Life Story Short-Form and Life Story Narrative sections).

## Discussion

### Principal Findings

This co-design process found that integrating life stories into the acute care setting for patients with dementia or at risk of delirium is a highly acceptable intervention to patients, care partners, and the IDT. Stakeholders were unanimously supportive of the idea of using a life storytelling intervention in acute care. They described potential benefits to integrating life storytelling into acute care including humanizing patients, building patient- and care partner–IDT relationships, sharing information about patients with dementia or at risk of delirium, and addressing potential biases based on age, race, or other factors. Stakeholders described potential challenges including the necessity to focus on acute patient needs, short lengths of stay, the need for IDT member buy-in, and patient capacity and privacy concerns.

The existing MemoryWell life storytelling intervention, previously used in long-term care in the form of 500- to 700-word narratives providing care teams information about patient’s personal histories and preferences, was adapted to meet stakeholder preferences and to best fit the acute care setting needs. These modifications include focusing the life story interview guide to cover stakeholder-selected topics (eg, significant focus on social support), the creation of a brief, bullet point summary to help clinicians quickly learn information most applicable to caring for acute needs and discharge planning, and a multipronged approach to story EHR integration. Stakeholders shared their perspectives on ideal timing for life story interviews, including interviewing the patient as soon as possible while ensuring clinical workflow and medical procedures are not interrupted.

The study resulted in the creation of a co-designed life storytelling interview guide and format for EHR integration and wireframes depicting future EHR integration, and ideas to fit a life storytelling intervention into acute hospital workflows, to be piloted in a future study. A future pilot is needed to explore the actual implementation of the co-designed intervention, including logistics, usability, and impact on the patient, IDT, and care partner outcomes.

### Comparison With Prior Work

This study is the first to our knowledge that has co-designed a life storytelling intervention for patients with dementia and at risk of delirium in the hospital setting. Our findings mirror previous patient narrative interventions aimed at sharing patients’ personal histories, values, and beliefs with their care teams in other clinical settings in terms of acceptability, challenges to implementation, and anticipated benefits. Though some life story work has focused on older adults with dementia, none has focused specifically on those with or at risk of delirium. Life story work has most often been implemented in long-term care settings [24,39-41] and has been acceptable among staff, patients, and care partners [20,21,24,39-41]. Evaluations of the My Life My Story program at the Veterans Administration have found that most staff read narratives [21]. One study involving patients with serious illness and without dementia in acute care found a narrative intervention to be acceptable to patients and bedside nurses [20].

The potential challenges to life story implementation uncovered in this study, including barriers to care team engagement, suitability to the acute care environment, and concerns for patient capacity and privacy are similar to previous studies [20]. One feasibility study of life stories for older adults with dementia in the United Kingdom on acute care National Health Service wards found similar time barriers and a need for prioritizing acute needs [42]. Approaches to the involvement of care partners in storytelling varied by study; some studies caution against using care partners to tell patients’ stories [43], while others acknowledge the need for care partner participation dependent on patients’ cognitive status [24]. We found that to co-design a life storytelling intervention for this population in acute care, care partners must be involved for patients with dementia and those at risk of delirium.

The anticipated benefits in our study are similar to anticipated and actual benefits in previous work, including improving individually tailored care; promoting relationships and communication among patients, care partners, and care teams; and particular benefits for people with dementia or risk of delirium, including serving as a communication tool for patients who are unable to communicate for themselves. Life story studies involving older adults with dementia have also shown that stories humanize patients, improve patient- and care partner–care staff relationships, and aid patient care [42-44].

### Limitations

This study has several limitations. The primary limitation is that it was difficult to recruit and interview patients with dementia or at risk of delirium while they were in the hospital, due to acute illness, consistent clinical interventions, and limited cognitive capacity. Another limitation was the lack of cultural diversity patients recruited, as we were only able to recruit English-speaking patients and care partners. We did allow for English-speaking care partners to participate even if the patient did not speak English, to include perspectives from families with non-English speakers. Our recruitment was limited to 1 unit at 1 hospital and used only the APeX EHR, which means findings may not be generalizable to other EHRs, which could be a barrier to expanding the intervention. Finally, we experienced challenges in recruitment as the ACE Unit beds were delegated to patients who tested positive for COVID-19 of all ages during the study. As the pandemic numbers waxed and waned, this limited the number of available beds for older eligible patients, thus restricting the pool of eligible patients to recruit for our study.

### Conclusions

In this study, we have co-designed with patients, care partners, and interdisciplinary team members, an adapted MemoryWell life story intervention for patients with dementia or at risk of delirium in an acute care hospital setting. This study highlights the anticipated impact of life stories for improving person-centered care in this setting, which often has limited resources and tools to do so. Future studies should be conducted to assess implementation feasibility and if these anticipated impacts on quality clinical and person-centered care can be made.

## Appendix

Multimedia Appendix 1 Study-specific qualitative interview guides used in stage 1.

## Abbreviations

ACE: Acute Care for Elders
ACP: Advance Care Planning
AER: Apex Enabled Research
EHR: electronic health record
IDT: interdisciplinary care team
SNF: skilled nursing facility
UCSF: University of California San Francisco
