# Supplementary material for: Developing a Life Story Intervention for Older Adults With Dementia or at Risk of Delirium Who Were Hospitalized: Multistage, Stakeholder-Engaged Co-Design Study
Source: JMIR Aging. 2024 Sep 27;7:e59306. doi: 10.2196/59306 (PMC11470218; doi:10.2196/59306)
Supplement: Multimedia Appendix 1 [file aging_v7i1e59306_app1.pdf]

Appendix 1. Qualitative Interview Guides:

Interdisciplinary Care Team Interview Guide

Preamble: MemoryWell employs professional writers to interview patients with cognitive impairment and write their life story. We have used our patient life stories in other settings and want to gain your perspective on if we could adapt these life stories to be useful in the acute care setting at UCSF. The life story would be shared with care teams in a digital format/EHR with the goal of helping them get to know patients better.

1. Life story interest
    a. *First, we want to gauge your interest in integrating life stories in acute care.* Do you think capturing patient life stories is important? Why/why not?
2. Life story content
    a. What kind of information about patients' lives would you want to see in the life story?
        i. Life stages
            1. Childhood: where they grew up, family background, challenges and setbacks
            2. Young adulthood: college, military, achievements
            3. Adulthood: marriage, children, career
        ii. Social/spiritual/activities
            1. Hobbies, pastimes, favorite books, movies, tv, music
                a. How these have changed over time
            2. Favorite foods, recipes
            3. Friends and family
            4. Pets
            5. Religion/spirituality
        iii. Legacy/hopes/achievements
            1. What they are proud of
            2. What their hopes and wishes are
            3. What would make them more comfortable during their stay in the hospital
        iv. Social determinants of health: housing, family/friend support, food, transportation
    b. What are two-three things you would definitely want to know in someone's life story?
3. Potential outcomes of life stories
    a. How would knowing the information from life stories impact your care?
    b. Do you think life stories would be beneficial for:
        i. Decision making for individual patients?
        ii. Patient experience?
            1. Are there certain patients who would benefit the most from this?
        iii. Family members?
        iv. Clinical environment/hospital?
            1. How would it be beneficial?

        a. Categories to prompt:
            i. Clinical/improved medical outcomes
            ii. Provider outcomes (e.g. job satisfaction)
            iii. Patient provider relationship
            iv. Family provider relationship
            v. Hospital workflow
            vi. Finances
            vii. Care transitions
            viii. Any other outcomes?
    c. Are there certain types of providers this might be most beneficial for?
    d. Why would a health system want to invest in this?
    e. Why wouldn't stakeholders (care providers, patients/families, health system) want to incorporate patient life stories into the acute care setting?
        i. Do you see any potential drawbacks or harms in incorporating life stories into acute care?
            1. Emotional burden, time burden, challenges with setting and population
    f. Tell me about a time when knowing a patient's story was important to you?
    g. How did it help or change your experience with the patient?
    h. What were some of the most important pieces of the story for you?
    i. What do you think are some of the reasons stories are captured more regularly?
4. Life story capture
    a. Optimal timing
        i. When should life stories be captured in the acute care hospital setting? Are there times in a patient's day or hospital stay where interviewing them would be inconvenient for the patient or staff?
            1. Timing in relation to when patients are admitted
            2. Time of day
            3. Other workflow considerations
        ii. What factors in a patient's hospital stay would make taking a life story difficult that you would like MemoryWell to be aware of?
    b. Best way to capture patient life story interviews in clinical environment
        i. How should life stories be captured in the acute care setting?
            1. Mode: in person, zoom, phone
        ii. What do you think about having MemoryWell collect life stories?
            1. What are the benefits of having someone outside of the care team collect the story?
            2. What are the drawbacks?
5. Life story implementation
    a. Drivers
        i. We estimate that it would take 1-2 minutes to read, do you think you would do that?
        ii. What would encourage you to read the life stories?

        iii. (After showing a sample story) Could you mark up, change or suggest any changes you think would make it easier to use this?
        iv. If we offered something more brief, would you want a paragraph or a few bullets?
        v. How much time (seconds to minutes) would you spend reading a life story?
        vi. What would make this worth your time?
        vii. When in your workday would you read this?
            1. When you first see the patient? Everytime you see them?
            2. Would you read it before or after meeting them for the first time?
    b. Barriers to incorporation of life stories into their care routines
        i. What are some of the reasons why you wouldn't use this?
        ii. What kinds of changes in workflow would need to be made to enable you to take the time to read life stories?
        iii. Are there any kinds of organizational culture or management changes that would need to occur to implement this?
    c. What kind of training or support would you want to have around life stories? Do you think this would make it more likely for you to use the life story?
        i. Technical support
        ii. Guidance for incorporating life story knowledge into care
        iii. Communication
            1. Recognizing and responding to difficult issues, maintaining boundaries, sensitivity
        iv. What would be the best format for this training? Zoom? Written materials?
        v. When would you want to have the training? Are there times when the care team is meeting or it would be easy to build into your routine?
6. Input on EPIC options
    a. Could you sketch out or describe when in your day you think you would review a story? What are you typically doing at that time? Where (What screen) in APeX might you already be?
    b. Where would you be most likely to see this in EPIC?
    c. How often do you see yourself accessing this?
    d. What would help incorporate this into clinical workflow?
    e. How would you want to be alerted that it is uploaded?
    f. What would be good metrics for assessing usage of the life story in EPIC?
    g. Do you hand this off to the next person–would you use some of this information?
    h. Is this information you would want to share with next site of care? Nursing home/PCP
        i. Do you see benefits in this?
        ii. Do you see drawbacks in this?

Care Partner and Patient Interview Guide

Preamble: UCSF is working with professional writers who interview patients and write their life story. We are working to create a life storytelling program for patients in the hospital. We want to learn what you think of this idea. The life story would be based on an interview with a professional writer, then it would be shared with the patient, their family, and with the doctors and nurses at UCSF through the electronic medical record.

(Note: wording was changed to address the patient directly in patient interviews)

1. Life story interest
   We want to learn what you think about creating a program to share patient life stories in the hospital and if you would be interested in this kind of program.
   a. Degree of interest in life stories
      i. If you were given the opportunity, Would you be interested in sharing [patient name]'s life story with the care team (for ex. doctors, nurses, social workers, case managers, patient care assistants and chaplains) at this hospital?
      ii. Why/why not?
   b. Do you think sharing patients' life stories is important? Why/why not?
2. Life story content
   a. What would you like to share in [patient name]'s life story?
   b. What do you think [patient name] would most want to share?
      i. Why would you want to share these specific kinds of details?
   c. Here are some topics patient life stories often include, would you be comfortable sharing these kinds of details?
      1. Life stages
         a. Childhood: where they grew up, family background, challenges and setbacks
         b. Young adulthood: college, military, achievements
         c. Adulthood: marriage, children, career
         d. Retirement/later years
      2. Social/spiritual/activities
         i. Hobbies, pastimes, favorite books, movies, tv, music
            1. How these have changed over time
         ii. Favorite foods, recipes
         iii. Friends and family
         iv. Pets
         v. Religion/spirituality
      3. Legacy/hopes/achievements
         i. What they are proud of
         ii. What their hopes and wishes are
         iii. What would make their lives more comfortable
         iv. Life experiences you would like to have
         v. What matters most to you as you think of the future

            4. Social determinants of health: housing, family/friend support, food, transportation
    d. Is there anything you/[patient name] would not want to share?
3. Life story impact:
   We are interested in your thoughts on how sharing someone's life story with their doctors and nurses could change their experience at the hospital.
    a. Do you think if [patient name]'s doctors and nurses and social workers knew more about [patient name] they would treat [patient name] differently?
        i. E.g. how you communicate, decision making
    b. How would it impact your relationship with [patient name]'s doctors and nurses as a caregiver?
    c. Do you think the process of putting together [patient name]'s life story would impact your own relationship with [patient name]?
4. Life story process input
   We would like to learn more about how we can make the life story process better for patients and families. This includes the interview itself, creating the story, and sharing it with doctors and nurses.
    a. Optimal timing/best way to capture life story
        i. If given the opportunity, Is this something that you would be willing to do while they are in the hospital?
        ii. When would you want to complete a life story interview? (Time of day, time during stay)
        iii. Would you prefer to complete the interview in person, over the phone or on zoom?
    b. What else would make it easier for [patient name] to share their story?
    c. Input on delivery options
        i. Would you be interested in accessing the life story on MyChart if the option was available?
        ii. Would you need assistance accessing this?
        iii. Is there any other way you would want to access the life story? Would you like a printed copy? Would you want it to be emailed to you? Or have a link texted to you?
5. *We want to make sure we understand any potential concerns families or patients may have about the process of creating the life story and sharing their life story.*
    a. What concerns would you have about sharing [patient name]'s life story with their doctors and nurses?
    b. Would you feel comfortable with [patient name] participating in the life story interview without a family member and/or caregiver present? Why/why not?

   To create the life story, a professional writer would interview [patient name] and write up their life story. This means someone outside of the UCSF care team at this hospital would be completing this process. The UCSF care team would get your permission to put the writer in touch with you.
    c. Would you be comfortable telling [patient name]'s life story to someone who does not work at the hospital? Why/why not?

When a life story is shared in the electronic medical record, it is there permanently and your whole care team can see it. It also may be shared through [patient name]'s electronic record to other healthcare providers. We would like to learn about how you feel about this.

d. Would you be comfortable with all of [patient name]'s providers at this hospital being able to read their story? Why/why not? Are there some healthcare providers at this hospital who you wouldn't want to see this?
e. Do you see a benefit in the story moving with [patient name] to different care facilities/other doctors or hospitals in the future?
    i. Or sharing it with their primary care provider?
    ii. Given that the life story would be a part of the permanent medical record, what concerns do you have about this?
f. Would you want to share the life story with other people in your/[patient name]'s life outside of the hospital?
